# Overall survival of patients with thyroid cancer in Martinique (2008–2018)

**DOI:** 10.1186/s12885-023-11072-1

**Published:** 2023-08-10

**Authors:** Lyvio Lin, Thierry Almont, Murielle Beaubrun, Jonathan Macni, Aimée Pierre-Louis, Audrey Zabulon, Ciprian Draganescu, Lucien Lin, Nadia Sabbah, Moustapha Drame, Jacqueline Veronique-Baudin, Clarisse Joachim

**Affiliations:** 1 CHU Martinique, UF1441 Registre Des Cancers de La Martinique, Pôle de Cancérologie Hématologie Urologie, Fort de France, Martinique France; 2 CHU Martinique, UF3596 Recherche en Cancérologie, Pôle de Cancérologie Hématologie Urologie, Fort de France, Martinique France; 3 Pôle Cardiovasculaire Thoracique Maladies Métaboliques Et Endocriniennes Néphrologie Hémodialyse, CHU Martinique, UF2151 Service d’endocrinologie, Fort de France, Martinique France; 4 CHU Martinique, UF1431, Médecine Nucléaire, Pôle Imagerie Médicale, Fort de France, Martinique France; 5 Pôle Cardiovasculaire Thoracique Maladies Métaboliques Et Endocriniennes Néphrologie Hémodialyse, CHU Martinique, UF3540 Service d’endocrinologie, Fort de France, Martinique France; 6 Service d’Endocrinologie, Centre Hospitalier de Cayenne, Guyane, France; 7 CHU de La Martinique, UF3163, Unité de Soutien Méthodologique À La Recherche (USMR), Délégation À La Recherche Clinique Et de L’Innovation (DRCI), Fort de France, Martinique France; 8 Hôpital Albert Clarac, Avenue PASTEUR, C.S 90632 97200 Fort de France, France

**Keywords:** Thyroid cancer, Survival analysis, Prognostic factors, Cancer registry

## Abstract

**Background:**

Thyroid cancer (TC) overall survival at 5 years was estimated at 97% in mainland France over 2010–2015. Its prognosis is known to be affected by patient age, tumor histology, size, and extension. This study aims to describe overall survival of thyroid cancer patients diagnosed between 2008 and 2018 in Martinique.

**Methods:**

We included in this retrospective analytical study all patients who were diagnosed with thyroid cancer. An overall survival analysis at 1, 3 and 5 years of thyroid cancer patients diagnosed in Martinique from 2008 to 2018 was conducted. Prognostic factors associated with survival have been identified. Stage at diagnosis and patterns of care among thyroid cancer patients were analyzed.

**Results:**

A total of 323 thyroid cancer patients were registered between 2008 and 2018. Papillary carcinomas represented 83% of diagnoses. Local stage or locally advanced invasion was found in 264 (88%) patients. 221 Multidisciplinary Teams reports files were reviewed. The overall survival observed in this population is 97% [93–99] at 1 year, 93% [88–97] at 3 years and 91% [85–95] at 5 years. Anaplastic, poorly differentiated and medullar tumors had lower survival rates at 5 years (39% [13–65]) compared to papillary tumors (93% [89–96]). We found that metastatic stage at diagnosis (HR = 3.1[1.3–7.6]; *p* = 0.01) and tumor size > 3 cm (HR = 2.7 [1.1–6.3]) were independent prognostic factors for OS in our population.

**Conclusions:**

The survival rates of thyroid cancer in Martinique are comparable to those observed in France.

## Background

The GLOBOCAN 2020 estimates indicate that there were 19.3 million new cases of cancer and almost 10 million deaths from cancer worldwide in 2020 [1]. In the Latin America and Caribbean (LAC) region, 1.5 million new cancer cases and 700,000 deaths were estimated annually accounting for 7.6% of all cases worldwide.

In the LAC region, Martinique displayed, after Uruguay the second highest incidence rates for all cancers, both sexes combined; with an estimated age-standardized incidence of 248.7 per 100,000 person-year [2].

Thyroid cancer was responsible for 586,000 cases and 43,646 deaths worldwide, ranking in 9^th^ place for incidence in 2020. In LAC region it ranks 6^th^ with 63,368 cases and 4,406 deaths. A rising incidence of thyroid cancer is noticed since the early 1980s, with a low mortality that remains stable or is even declining [1].

This disease is most common in developed countries, notably in France [3]. In 2018, an estimated total of 10,665 cases, with 2,600 men and 8,065 women, were diagnosed with thyroid cancer in mainland France. The estimated number of deaths from thyroid cancer was 159 in men and 386 in women in mainland France in 2018.

Net survival at 5 years for persons diagnosed with thyroid cancer between 2010 and 2015 was estimated at 93% for men and 97% for women [4]. Patient age, sex, tumor histology, size, and metastatic extension are consistent prognostic factors of the disease in national and international studies [1, 4].

Martinique is an overseas department of France; this West Indies Island belongs to the Caribbean region. It covers an area of 1128 km^2^ with 354,800 inhabitants as of 2020 [5].

Since 1983, the Martinique Cancer Registry (MCR) collects all cancer cases in Martinique, for solid tumors and hematological malignancies. This population-based cancer registry has a high-level of data quality, guaranteed by data quality assessment performed by national and international agencies for cancer control. Martinique has the lowest overall incidence for all cancers, compared to French Guyana and Guadeloupe. However, Martinique has the highest number of thyroid cancer diagnosis with an average cumulate of 30 cases, 6 men and 24 women, per year in 2007–2014 [6].

This study aims to describe overall survival of thyroid cancer patients diagnosed between 2008 and 2018 in Martinique.

## Methods

### Study population

The Martinique Cancer Registry performs continuous and exhaustive recording of all new TC cases occurring in the population resident in Martinique, regardless of where the diagnosis or the treatment takes place. Thanks to this population-based cancer registry, all cancer cases are reported in the cancer database. We included in this retrospective analytical study all patients who were diagnosed with thyroid cancer (ICD10: C73) between 2008 and 2018.

### Data collection

Data were included in the MCR database according to the international standards laid down by the International Agency for Research on Cancer (IARC), taking to account the French FRANCIM network, and the recommendations of the European Network of Cancer Registries (ENCR). The MCR Registry procedures have the approval of the French National authority for the protection of privacy and personal data. The registry is cooperating with different local organizations to ensure an exhaustive data collection process. Data Quality check was performed in accordance with international recommendations for cancer registries.

We extracted from the MCR database the socio-demographic variables: sex, birth date, zone of residence at diagnosis. Clinical variables analyzed in this study were vital status, tumor size, histology, stage at diagnosis (TNM), date of diagnosis, date of last news or death. Clinical stage at diagnosis was classified following American Joint Committee on Cancer (AJCC)/TNM risk score from I to IV following 7^th^ edition scoring. Addition staging was performed dividing patients into locally advanced (T1-T4 N0M0), versus node (N + /M-), and metastatic group (M +).

Additional variables were extracted from Multidisciplinary Teams (MDT) reports and medical records archived at the University Hospital of Martinique. The following variables were: modality of cancer discovery, tumor site, surgery (lobectomy or total thyroidectomy), lymph node dissection, use of radioiodine, immunotherapy or chemotherapy. The availability of numerous archived digital MDT reports allowed us to largely implement our database with therapeutic characteristics.

We collected data regarding deaths from the French epidemiological center on medical causes of death. These data were obtained from the French National Institute of Health and Medical Research (CépiDc, Inserm: http://www.cepidc.inserm.fr/site4/), ensuring completeness of death information. Research of vital status is based on hospital and medical records; administrative databases are also used for data analysis. Vital status updates and corrections are made continuously every year.

### Statistical analysis

Patient characteristics are presented as mean ± standard deviation for quantitative variables, and as number (percentage) for all qualitative variables.

Age was categorized into three age groups: less than 45 years, 45 to 65 years, more than 65 years.

Four groups were presented for analysis of the zone of residence: Center, North-Atlantic, North-Caribbean, and South.

Two periods of analysis were used for this study: 2008–2012 and 2013–2018.

Overall survival (OS) with 95% confidence interval (CI) as the time from the date of diagnosis to the date of death from any cause was calculated. All thyroid cancer patients were censored at the date of last follow-up, or at the cut-off date of January 1, 2022 when the patients were alive at that date. We used the Kaplan–Meier product-limit method to estimate the proportion of survivors over time for univariate survival analysis. The log-rank test was performed to assess the statistical differences of the observed survival curves by each socio-demographic and clinical variable. Prognostic factors for OS were assessed using a multivariable Cox model for censored. Variables with a *p*-value < 0.20 in the Univariate analysis were included in the model for multivariable analysis. A *p*-value < 0.05 was considered statistically significant. All our analyses were performed using SAS version 9.4. (SAS Institute Inc., Cary, NC, USA).

## Results

A total of 323 thyroid cancer patients were registered in the MCR between January 1st 2008 and December 31th 2018, with 158 on the 2008–2012 period and 165 on the 2013–2018 period.

Women represented the majority of cancer cases with 263 patients (81%); 60 patients (19%) were men. In our study, 137 (42%) patients were living in the center, 88 (27%) in the South, 79 (25%) in the North Atlantic and 19 (6%) in the North Caribbean. Socio-demographic variables are presented in Table 1.

**Table 1:** Socio-demographic characteristics among patients with thyroid cancer according to age at diagnosis (*N* = 323)

	**All cases**		** < 45**		**[45–65]**		** > 65**	
	n	%	n	%	n	%	n	%
	323	100	88	27	155	48	80	25
**Period**								
2008–2012	158	49.0	49	56.0	81	52.0	28	35.0
2013–2018	165	51.0	39	44.0	74	48.0	52	65.0
**Sex**								
Male	60	19.0	10	11.0	29	19.0	21	26.0
Female	263	81.0	78	89.0	126	81.0	59	74.0
**Zone of residence**								
Center	137	42.0	43	49.0	63	41.0	31	38.0
North-Atlantic	79	25.0	18	21.0	42	27.0	19	24.0
North-Caribbean	19	6.0	3	3.0	5	3.0	11	14.0
South	88	27.0	24	27.0	45	29.0	19	24.0

In our population, papillary carcinomas represented 83% of diagnoses with 269 cancer cases, 40 (12%) for follicular carcinomas, 5 (2%) for medullary and finally 9 (3%) for anaplastic and poorly differentiated carcinomas.

The average tumor size was 2.21 cm, with a minimum of 5 mm and a maximum of 13 cm. Microcarcinomas (< 1 cm) were the most frequent type with a third of the population (*n* = 107; 37%); followed by intermediate size tumors, from 2 to 4 cm, representing 24% of cases (*n* = 70). Small tumors, from 1 to 2 cm were found in 63 (22%) patients, while large tumors, with diameter exceeding 4 cm, were found in 48 (17%) patients. Local stage or locally advanced invasion was found in 264 (88%) patients. Lymph node invasion was shown in 26 (9%) patients while metastatic stage at diagnosis was found in only 10 (3%) patients. AJCC staging showed mostly stage I cancer with 202 (67%) patients, stage II and III representing a total of 73 (24%) patients, while stage IV was found in 25 (8%) patients. Clinical variables are presented in Table 2.

**Table 2:** Clinical characteristics among patients with thyroid cancer according to age (*N* = 323)

	**All**		**< 45**		**[45–65]**		**> 65**	
	**N**	**%**	**N**	**%**	**N**	**%**	**N**	**%**
	323	100	88	27	155	48	80	25
**Histology**								
Papillary	269	83.3	77	87.5	129	83.2	63	78.7
Follicular	40	12.4	10	11.4	23	14.9	7	8.8
Medullar	5	1.5	-	-	3	1.9	2	2.5
Anaplastic or poorly differentiated	9	2.8	1	1.1	-	-	8	10
**Tumor size**								
< 1 cm	107	37.1	30	37.0	51	37.5	26	36.6
[1-2 cm]	63	21.9	15	18.5	39	28.7	9	12.7
[2-4 cm]	70	24.3	25	30.9	28	20.6	17	23.9
> 4 cm	48	16.7	11	13.6	18	13.2	19	26.8
Unknown	35	-	7	-	19	-	9	
**Clinical staging**								
Local	264	88.0	72	84.7	130	91.5	62	84.9
Lymph node reached	26	8.7	10	11.8	7	4.9	9	12.3
Metastatic	10	3.3	3	3.5	5	3.5	2	2.8
Unknown	23	-	3	-	13	-	7	-
**AJCC staging**								
I	202	67.3	82	96.5	86	60.6	34	46.6
II	35	11.7	3	3.5	20	14.0	12	16.4
III	38	12.7	-	-	24	16.9	14	19.2
IV	25	8.3	-	-	12	8.5	13	17.8
Unknown	23	-	3	-	13	-	7	-
**Discovery**								
Symptomatic	16	9.4	2	4.4	9	11.5	5	10.6
Goiter	88	51.8	27	60.1	33	42.3	28	59.6
Ultrasonography	58	34.1	15	33.3	30	38.5	13	27.7
Endocrinopathy	8	4.7	1	2.2	6	7.7	1	2.1
Unknown	153	-	43	-	77	-	33	-
**Neoplasic location**								
Isthmus	7	3.7	1	1.8	2	2.4	4	8.2
Right lobe	71	37.4	23	40.4	31	36.9	17	34.7
Left lobe	61	32.1	20	35.1	24	28.6	17	34.7
Multiple	51	26.8	13	22.7	27	32.1	11	22.4
Unknown	133	-	31	-	71	-	31	-
**Invasive**								
Yes	61	36.1	20	42.6	25	32.5	16	35.6
No	108	63.9	27	57.4	52	67.5	29	64.4
Unknown	154	-	41	-	78	-	35	-

Out of the 323 patients, 221 (68%) Multidisciplinary Team reports have been found and reviewed; a total of 217 patients underwent surgery. A partial resection of the gland (lobectomy) was reported for 60 patients. A complete thyroidectomy was decided for 157 patients. Lymph node resection (LNR) was performed for 59 patients.

Among 72/107 patients with microcarcinomas, 18 had a lobectomy and 52 had a total thyroidectomy.

A total of 134/221 patients received radioiodine. We described 3 therapeutic profiles in our population: 74 patients had surgery-only profile, 96 patients received radioiodine or LNR in addition to surgery, 51 patients had both LNR and radioiodine in addition to surgery as their cancer treatment. All data are presented in Table 3.

**Table 3:** Therapeutic characteristics among patients with thyroid cancer according to age at diagnosis (*N* = 323)

	**All**		** < 45**		**[45–65]**		** > 65**	
	**N**	**%**	**N**	**%**	**N**	**%**	**N**	**%**
	323	100	88	27	155	48	80	25
**Surgery**								
Lobectomy	60	27.6	20	30.8	27	27.3	13	24.5
Thyroidectomy	157	72.4	45	69.2	72	72.7	40	75.5
Unknown	106	-	23	-	56	-	27	-
**Lymph node resection (LNR)**								
Yes	59	27.6	20	30.8	20	20.8	19	35.8
No	155	72.4	45	69.2	76	79.2	34	64.2
Unknown	109		23		59		27	
**Radioiodine (RI)**								
Yes	134	63.5	39	60.9	63	64.3	32	65.3
No	77	36.5	25	39.1	35	35.7	17	34.7
Unknown	112	-	24	-	57	-	31	-
**Therapeutic profile**								
Surgery only	74	33.5	23	34.8	35	34.7	16	29.7
Surgery + LNR or RI	96	43.4	25	37.9	47	46.5	24	44.4
Surgery + LNR + RI	51	23.1	18	27.3	19	18.8	14	25.9
Unknown	102	-	22	-	54	-	26	-

Most thyroid cancers were diagnosed over a goiter (88 patients) or during a routine ultrasonography (58 patients). In total, 16 patients were diagnosed due to compressive symptoms (dysphonia, dysphagia, mediastinal compression) and 8 patients were diagnosed during exploration for another endocrinopathy. Regarding tumor site, 51 patients had multiple neoplasic location in the thyroid gland, 61 patients had their cancer located in the left lobe, 71 in the right lobe. Finally, 7 patients had their tumor located in the isthmus. Archived files reported no sign of local advancement in 108 tumors, invasive characteristics were found for 61 patients (Table 2).

### Overall survival

**Fig. 1:**
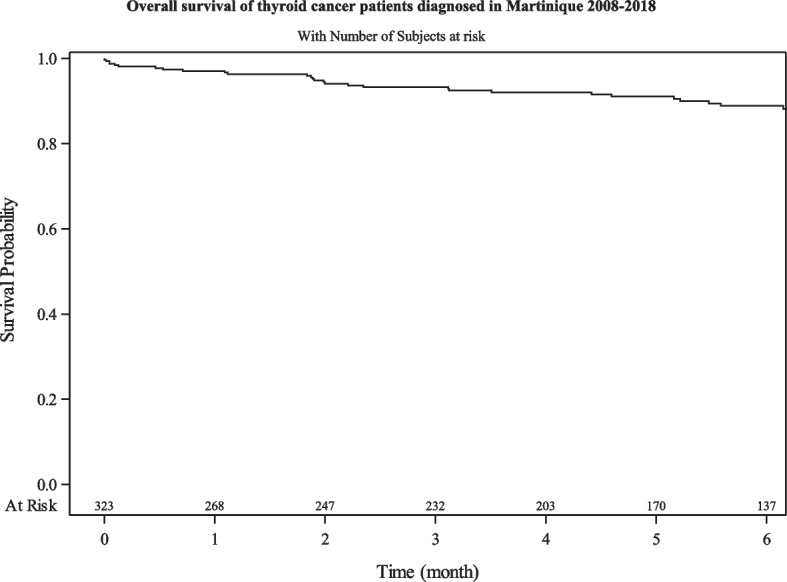
Analysis of overall survival in thyroid cancer patients

In our study, we observed an overall survival rate of 97% [93–99] at 1 year, 93% [88–97] at 3 years and 91% [85–95] at 5 years (Fig. 1). There was no significant difference in the overall survival rates between the 2008–2012 and the 2013–2018 periods. Overall survival rates are shown in Table 4.

**Table 4:** Overall survival in thyroid cancer patients in Martinique, 2008–2018

Characteristics	1 year % [95% CI]	3 years % [95% CI]	5 years % [95% CI]	Log-rank (*p*-value)
**All**	97.0% [93–99]	93.0% [88–97]	91.0% [85–95]	-
**Period**				
2008–2012	97.0% [92–99]	94.0% [88–96]	92.0% [87–96]	0.43
2013–2018	97.0% [93–99]	93.0% [87–96]	89.0% [80–94]	
**Zone of Residence**				
Center	96.0% [91–98]	94.0% [88–97]	91.0% [84–95]	0.70
North Atlantic	98.0% [96–100]	95.0% [86–98]	93.0% [83–97]	
North Caribbean	1	83.0% [48–96]	83.0% [48–96]	
South	96.0% [89–99]	93.0% [84–97]	91.0% [82–96]	
**Sex**				
Women	98.0% [95–99]	95.0% [91–97]	93.7% [90–96]	0.0002
Men	95.0% [85–98]	86.0% [73–93]	78.1% [62–88]	
**Age (years)**				
≤ 65	99.0% [96–100]	98.0% [95–99]	96.0% [93–98]	< 0.0001
> 65	92.0% [84–97]	79.0% [67–87]	75.0% [63–84]	
**Histology**				
Anaplastic, poorly differentiated or medullar	59.0% [28–81]	39.0% [13–65]	39.0% [13–65]	< 0.0001
Papillary or follicular	99.0% [96–99]	96.0% [92–97]	94.7% [91–97]	
**Stage**				
Local or locally advanced	99.0% [0.97–1]	97.0% [0.94–0.99]	95.0% [0.91–0.97]	< 0.0001
Metastatic	94.0% [0.78–0.98]	80.0% [0.61–0.91]	77.0% [0.57–0.88]	
**AJCC score**				
I—II – III	100.0% [97–100]	97.0% [94–98]	95.2% [91–97]	< 0.0001
IV	87.0% [66–96]	69.0% [46–84]	64.3% [41–80]	
**Tumor**				
≤ 3 cm	97.0% [93–98]	95.0% [91–97]	93.5% [89–96]	0.04
> 3 cm	99.0% [91–100]	89.0% [77–94]	82.2% [69–90]	
**Diagnosis due to compressive symptoms**				
Yes	87.0% [56–96]	70.0% [39–88]	60.7% [29–82]	< 0.0001
No	98.0% [95–99]	94.0% [91–97]	95.0% [89–98]	
**Therapeutic profile**				
Surgery only	1	96.0% [87–99]	94.0% [83–98]	0.15
Surgery + LNR or RI	97.0% [90–99]	94.0% [87–98]	91.0% [82–96]	
Surgery + LNR + RI	98.0% [86–100]	88.0% [73–95]	85.0% [69–93]	

**Fig. 2:**
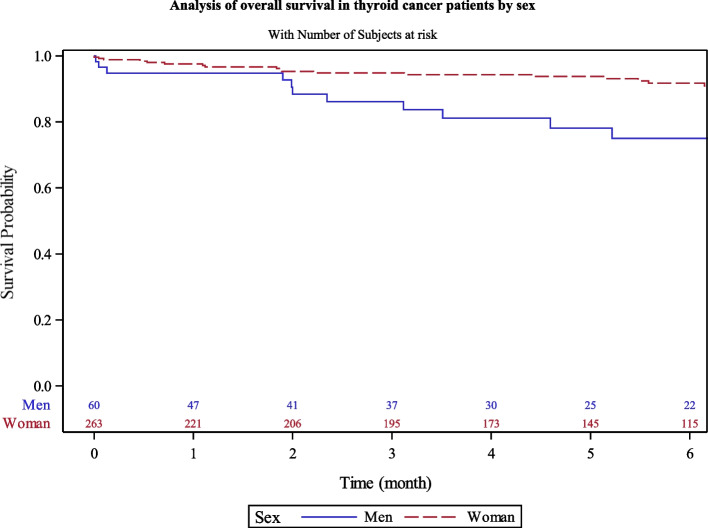
Analysis of overall survival in thyroid cancer patients by sex

**Fig. 3:**
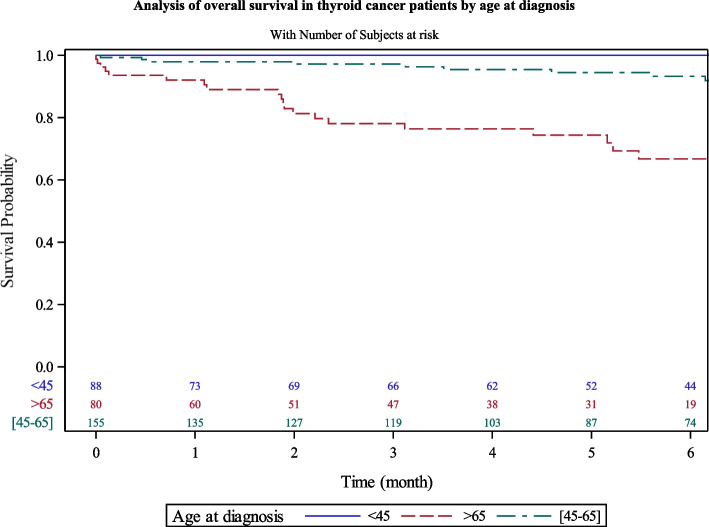
Analysis of overall survival in thyroid cancer patients by age at diagnosis

Men had lower overall survival rate (78.1% [62–88]) versus women (93.7% [90–96]) at 5 years (Fig. 2). Patients over 65 years had lesser survival rate at 5 years (74.9% [63–84]) than younger patients (96.3% [93–98]) (Fig. 3). The survival rates were also similar into the four territories of Martinique.

**Fig. 4:**
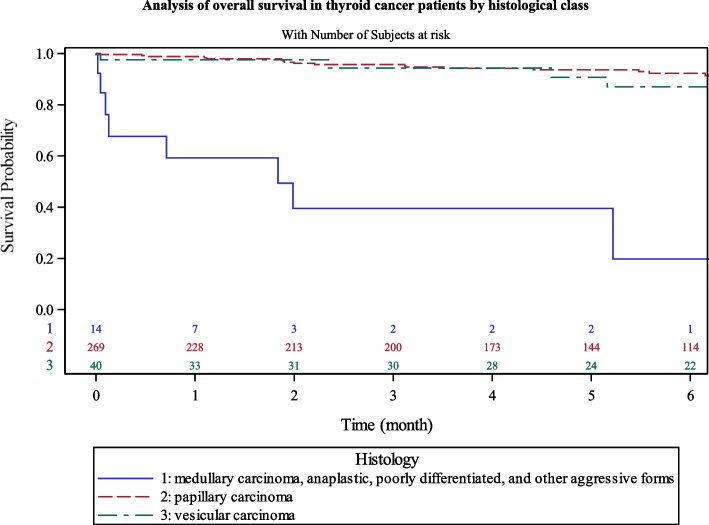
Analysis of overall survival in thyroid cancer patients by histological class

Anaplastic, poorly differentiated and medullar tumors had lower survival rates at 5 years (39% [13–65]) compared to papillary tumors (93% [89–96]) and follicular tumors (90% [74–97]) (Fig. 4). However, we had few cases of anaplastic or poorly differentiated carcinomas; the confidence intervals of our survival estimates were thus very wide. The difference between papillary and follicular carcinomas survival was not significant.

**Fig. 5:**
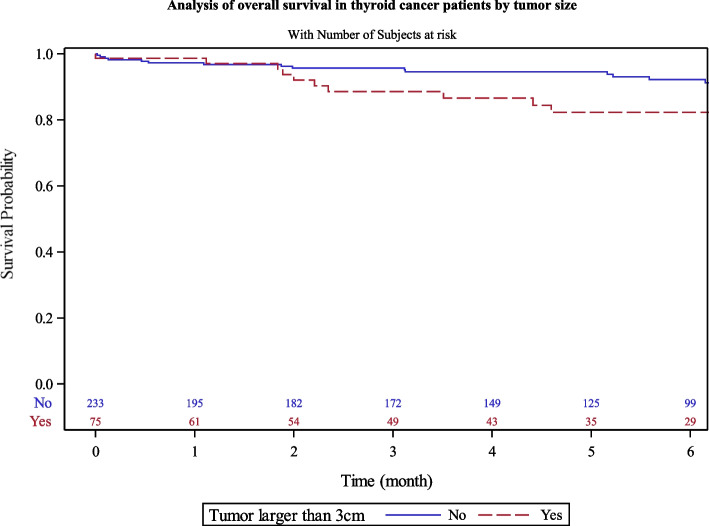
Analysis of overall survival in thyroid cancer patients by tumor size

Patient with tumor size > 3 cm had lower survival at 5 years (82.2% [69–90]) than those ≤ 3 cm (93.5% [89–96]) (Fig. 5).

**Fig. 6:**
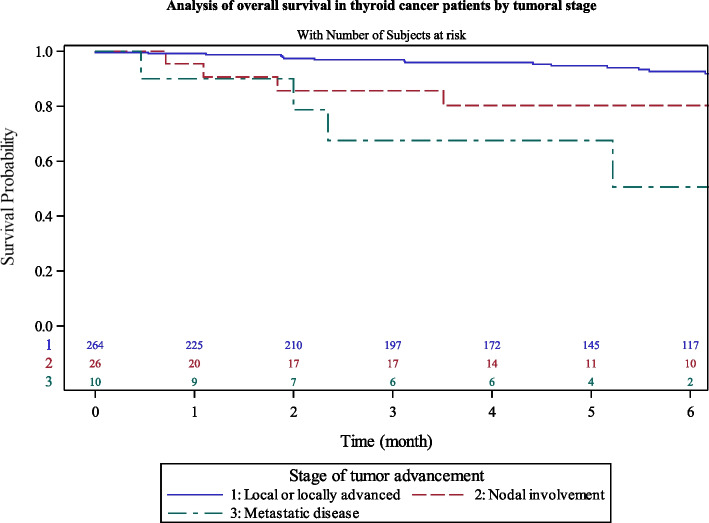
Analysis of overall survival in thyroid cancer patients by clinical stage at diagnosis

**Fig. 7:**
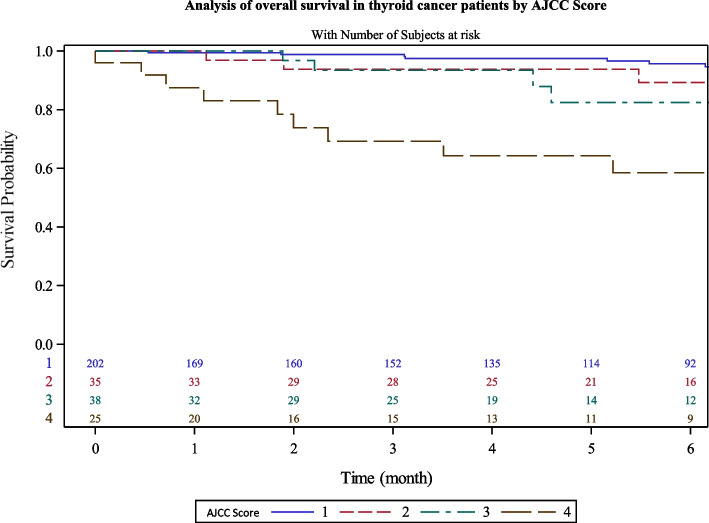
Analysis of overall survival in thyroid cancer patients by AJCC stage

We observed that patients with lymph node invasion or metastatic cancer had 5-years survival at 76.5% [57–88] while non-metastatic patient had a significant higher rate of 94.7% [91–97] at 5 years (Fig. 6). Patients with AJCC lower than IV had a 5 years-survival rate of 95.2% [91–97] compared to than with stage IV (64.3% [41–80]) (Fig. 7).

**Fig. 8:**
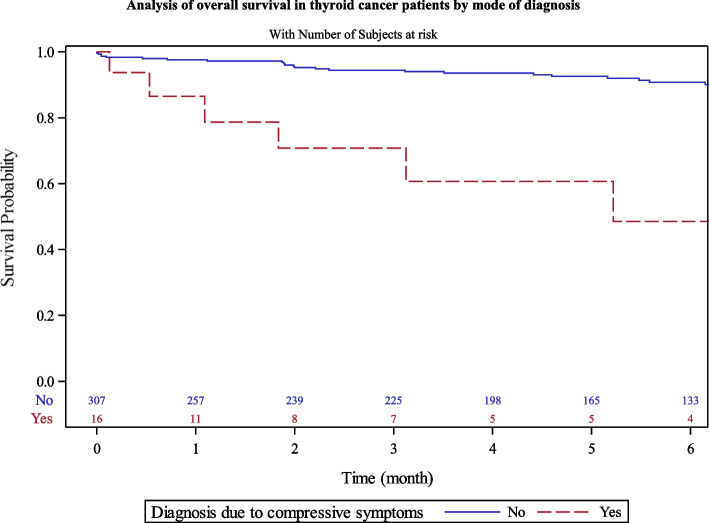
Analysis of overall survival in thyroid cancer patients by mode of diagnosis

Patients diagnosed due to cancer symptoms showed lower 5-year survival rate (60.7% [29–82]) than those diagnosed by ultrasonography screening, clinical goiter, or due to other endocrine exploration (95% [89–98]) (Fig. 8).

We found that metastatic stage at diagnosis (HR = 3.1[1.3–7.6]; *p* = 0.01) and tumor size > 3 cm (HR = 2.7 [1.1–6.3]) were independent prognostic factors for OS. We observed no significant difference according to histological type in our multivariable model (Table 5).

**Table 5:** Prognostic factors of thyroid cancer survival in Martinique, 2008–2018 (*n* = 323)

	Univariate HR [CI95%]	*p*	Multivariate HR [CI95%]	*p*
**Age (years)**				
≤ 65	1	< 0.001		
> 65	11.5 [5.6—23.8]			
**Stage**				
Local	1	< 0.001	1	0.01
Metastatic	4.9 [2.3 – 10.2]		3.1 [ 1.3 – 7.6]	
**Histology**				
Papillary or follicular	1	< 0.001	1	0.05
Anaplastic, poorly differentiated or medullar	14.3 [6.7—33.3]		4.5 [0.98 – 20.8]	
**Tumor size**				
≤ 3 cm	1	0.04	1	0.02
> 3 cm	1.96 [1.02—3.8]		2.7 [1.1–6.3]	
**AJCC**				
I or II or III	1	< 0.001		
IV	9.09 [ 4.5 – 20]			

## Discussion

This study is the first survival analysis of thyroid cancer patients in Martinique and in the Caribbean. It was supported by high-quality data from a population-based cancer registry with analysis of stage at diagnosis and socio-demographic characteristics. We showed that our patients had a very good prognosis, with a 91% 5-years survival rate in this territory over 2008–2018. The added value of this study was to provide patterns of care in thyroid cancer patients. Very few studies reported data regarding thyroid cancer survival in the LAC area; most studies provided insight on incidence or mortality data [7].

Thyroid cancer predominantly affects women; in Martinique 81% of thyroid cancer patients were women. There was a statistical difference between men and women survival with a better survival among women (94% OS at 5-years versus 78% in men). A national survival analysis on thyroid cancer was performed by the FRANCIM network of French population-based cancer registries for the 1975–2004 period. It showed higher survival rates regarding papillary carcinomas, and higher survival in women [8]. Nonetheless, this national study did not provide data regarding French overseas department. Furthermore, no stage at diagnosis data was available at that period. Another study was performed on the 1989–2018 period. The net 5-years survival in mainland France was estimated at 93% for men and 97% for women in 2010–2015. This national study compared 1-year and 5-years survival rates according to age at diagnosis, showing stability of survival until 70 years old in thyroid cancer patients [4]. In our study, OS remained > 90% at 5-years, displaying very good prognosis.

The histological repartition of thyroid cancer in Martinique displayed 83% of papillary, 12% of follicular, 2% of medullar and 3% of anaplastic and poorly differentiated carcinomas. Those data are consistent with national [4] and international reports [1, 2]. In our study the majority of our patients had a histology with a good prognosis (OS > 90%). In our 9 patients with anaplastic and poorly differentiated carcinomas, 8 patients were aged > 65 years at diagnosis. We observed 3-years OS of 39% in this group which was statistically different compared to papillary or follicular carcinoma (96% of 3-years OS).

We observed that 17% of our patients had a tumor size > 4 cm; Microcarcinomas represented 37% of our cases. In our survival analysis, according to tumor size, we observed a significant difference for tumor size > 3 cm; this group had lower survival with 5-years OS of 82% versus 93%.

Consistently with larger-scaled studies, we observed that metastatic stage at diagnosis (HR = 3.1[1.3–7.6]) and tumor size > 3 cm (HR = 2.7 [1.1–6.3]) were independent prognostic factors for OS. However, in our multivariable model, there was no significant difference according to histological type (HR = 4.5 [0.98 – 20.8]). Although anaplastic, poorly differentiated, and medullar tumors had statistically lower survival rates than other forms, cases were very rare in our population. This could have induced a lack of power in estimating the prognostic value of this factor.

Since the 1980s, rapid rises in incidence rates and comparatively stable mortality rates have been observed for thyroid cancer in much of the world [1, 8, 9]. Growing knowledge and diffusion of guideline for optimal medical care combined with early clinical detection contributed to lower the mortality associated to this disease. However, the rapid increase in all TC forms, and especially the papillary, can be attributed to broader use of ultrasonography, along with other diagnostic imaging modalities. Those tools led to a massive detection and diagnosis of subclinical, indolent lesions. Among women, overdiagnosis accounts for a large number of cases: 80% to 95% of newly diagnosed cases from 2008 to 2012 in the Republic of Korea, Belarus, China, Italy, Croatia, Slovakia, and France and from 50 to 70% in Denmark, Norway, Ireland, the United Kingdom, and Japan. The proportion attributable to overdiagnosis was approximately 10% lower in men than in women in each country [1]. Surgical treatment of indolent carcinomas implicates thyroid partial or complete resection, possible surgical complications, and lifelong hormonal substitution. In our study, 28% of our patients had lobectomy; 72% had a total thyroidectomy.

We also had 88% of patients with only local or locally advanced stage at diagnosis, with a 5-years OS of 95%. No equivalent study was found, using stage at diagnosis or AJCC scoring data. In fact, data collection of stage is very difficult to gather in countries lacking population-based cancer registries. Metastatic thyroid cancer had a 5-years OS of 77%. An AJCC stage IV was reported for 25 patients (8.3%). In our study, we also found a statistical difference regarding survival between AJCC score IV (63% of 5-years OS) versus other groups (95% of 5-years OS).

Thyroid cancer patients with diagnosis due to compressive symptoms had a lower 5-years OS (61% versus 95%). This underlines the necessity of early detection of thyroid cancer with ultrasonography and use of fine needle aspiration cytology. In order to tackle overdiagnosis and low benefits-risks ratio treatments issues, clinicians can rely on ulstrasonographic and cytological malignancy risk assessment tools, namely EU-TIRAD and BETHESDA classification, for exploratory and therapeutic decision.

Radioiodine was used in 63% of patients. This can affect fertility, especially ongoing pregnancy, as well as general radioprotection issue toward the patient and its surroundings [10]. This is important to confirm the cancer care project for patients, according to age at diagnosis, especially for women to take into account fertility before treatment.

Martinique hosted in 2018 and 2020 international consensus conferences, gathering European, American and Caribbean expertise in order to produce up-to-date guidelines, registered as “The Martinique Principles”, regarding interdisciplinary cooperation, therapy decision and use of radioiodine in thyroid cancer. The local impact of those new guidelines needs to be assessed in the future [11].

In the limits of our study, we faced a medium size population of 323 individuals with few deaths (39 events) occurring in the 11 observation years, linked to the moderate incidence and the very good prognosis of that disease. Those parameters restricted the statistical power of our analyses. Another important prognostic factors, i.e. biologic follow up data, as thyroglobulin, ACE or calcitonine, T4L and TSH levels, as well as EU-TIRADS and BETHESDA scores have been considered but weren’t available for collection in our analyzed cases.

The spatial distribution of thyroid cancer patients in Martinique followed that of the general population. However, we noticed that the North-Atlantic area that gathers 20% of the population (INSEE, 2019) represented 25% of thyroid cancer, whereas the South that gathers 32% of the populations holds only 27% of thyroid cancer cases. In Martinique, the University Hospital is located in the center of the island but also has a center in the North-Atlantic, where specialized clinical explorations of the thyroid gland are conducted. The North Atlantic area undergoes specific exposure to toxic environmental factors, such as sargassum and chlordecone. Variations in geographical distribution of thyroid cancer can therefore be hypothesized in terms of care services supply and environmental exposure.

Therapy data such as levothyroxin treatment could have been useful for explanation of thyroid cancer survival. Unfortunately, this data wasn’t available in the cancer registry database and would have required specific data collection using the integrated national health insurance database. This information could have been useful for a better assessment of observance and tolerance of that treatment. In the event of total thyroidectomy, hormonal substitution is systematic and lifelong. It is thus necessary for long-term patients’ survival and quality of life. Higher socioeconomic status is also known to be associated to early thyroid cancer detection. Those factors could be explored in further studies to assess their role for thyroid cancer survival.

## Conclusions

This is the first study describing prognostic factors of survival in thyroid cancer patients in Martinique. This study is consistent with national data and provides additional information on demographic, clinical and therapeutic characteristics that we can observe in mainland France, denoting similar disease expression in our population and proper medical care. Main prognostic factors were stage at diagnosis and tumor size. Net-survival analysis would also allow in the future new studies for better comparisons with Caribbean countries.

## Data Availability

The individual patient data that support the findings of this study are not publicly released. Data are however available from the corresponding author upon request and with permission of the Martinique Cancer Registry.

## Abbreviations

BMI: Body-mass index
CI: Confidence interval
CNIL: Commission Nationale Informatique et Libertés
DTC: Differentiated thyroid cancer
ENCR: European Network of Cancer Registries
EORTC: European Organization for Research and Treatment of Cancer
IARC: International Agency for Research on Cancer
ICD: International Classification Diseases
LAC: Latin America and Caribbean Region
LNR: Lymph Node Resection
MCR: Martinique Cancer Registry
MDT: Multidisciplinary Teams
MDT: Multidisciplinary Teams
OS: Overall Survival
RI: Radioiodine
TC: Thyroid Cancer
TNM: Tumor Node Metastasis
